# In vitro expansion of human sperm through nuclear transfer

**DOI:** 10.1038/s41422-019-0265-1

**Published:** 2019-12-18

**Authors:** Xiaoyu Merlin Zhang, Keliang Wu, Yuxuan Zheng, Han Zhao, Junpeng Gao, Zhenzhen Hou, Meiling Zhang, Jiaoyang Liao, Jingye Zhang, Yuan Gao, Yuanyuan Li, Lin Li, Fuchou Tang, Zi-Jiang Chen, Jinsong Li

**Affiliations:** 1 State Key Laboratory of Cell Biology, Shanghai Key Laboratory of Molecular Andrology, Center for Excellence in Molecular Cell Science, Shanghai Institute of Biochemistry and Cell Biology, Chinese Academy of Sciences, University of Chinese Academy of Sciences, 320 Yueyang Road, Shanghai, 200031 China; 2 Center for Reproductive Medicine, Shandong University, Jinan, Shandong 250001 China; 3 Beijing Advanced Innovation Center for Genomics, College of Life Sciences, Peking University, Beijing, 100871 China; 4 Biomedical Institute for Pioneering Investigation via Convergence, Ministry of Education Key Laboratory of Cell Proliferation and Differentiation, Beijing, 100871 China; 5 Peking-Tsinghua Center for Life Sciences, Academy for Advanced Interdisciplinary Studies, Peking University, Beijing, 100871 China; 6 Center for Reproductive Medicine, Shanghai Key Laboratory for Assisted Reproduction and Reproductive Genetics, Ren Ji Hospital, School of Medicine, Shanghai Jiao Tong University, Shanghai, 200127 China; 7 School of Life Science and Technology, Shanghai Tech University, 100 Haike Road, Shanghai, 201210, China

**Keywords:** Embryonic stem cells, Reprogramming

Dear Editor,

Mammalian haploid embryonic stem cells (haESCs) represent an ideal tool for genetic analysis due to the presence of only one set of genetic materials. HaESCs fall into two readily distinguishable groups based on their genome origins: parthenogenetic haESCs (PG-haESCs) that are derived from oocyte-originated parthenogenetic embryos and androgenetic haESCs (AG-haESCs) that are produced through sperm nuclear transfer. Both PG- and AG-haESCs are feasible for delineating genome function at cellular level in vitro. Importantly, AG-haESCs can be used as a sperm replacement and applied to deciphering gene function at organismal level when combined with CRISPR-Cas9 technology.^1^ However, while PG-haESCs have been generated from both rodent and primates,^2–5^ AG-haESCs can only be obtained in rodent to date.^6,7^

To investigate whether human sperm can be reprogrammed into haploid ESCs, we adopted a modified nuclear transfer protocol without cytoskeleton disruption^8^ to produce androgenetic (AG) embryos carrying only sperm genome (Fig. 1a; Supplementary information, Data S1). AG embryos could develop to blastocyst stage in vitro (Supplementary information, Fig. S1a, b, Table S1). From a total of 11 AG blastocysts, we derived 5 ESC lines under the standard human ESC culture conditions with 21% O_2_. However, haploid cells could not be enriched from all 5 lines in the initial cell sorting at passages 5–8 via fluorescence-activated cell sorting (FACS).

**Fig. 1:**
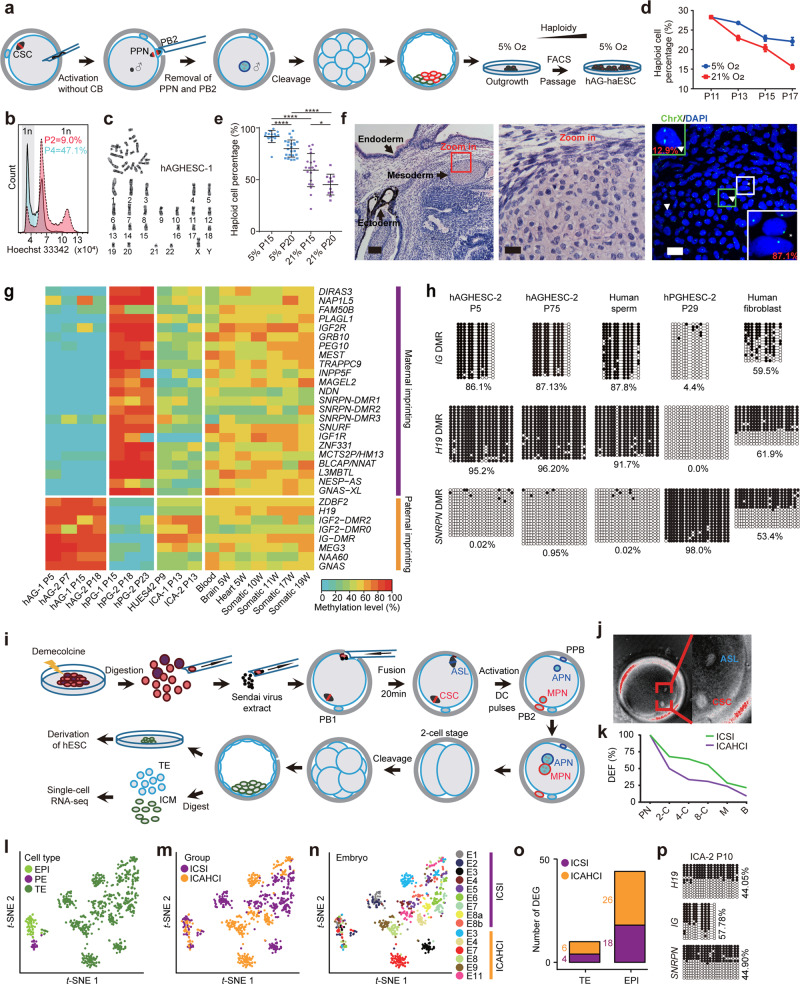
Derivation and application of human androgenetic haploid embryonic stem cells (hAG-haESCs). **a** Experimental diagram of deriving hAG-haESCs. CSC, chromosome-spindle complex; PPN, pre-pronucleus; PB2, second polar body; FACS, fluorescence-activated cell sorting; CB, cytochalasin B. **b** A stable hAG-haESC line, hAGHESC-2, was generated after one round of FACS enrichment of haploid cells. The percentage of haploid cells (G1 phase) was elevated from 9.0% (red) to 47.1% (cyan) after one round of enrichment. **c** G-banding analysis of hAGHESC-1 revealed the normal haploid complement of 23 chromosomes (22 + X). **d** The percentages of haploid cells in hAGHESC-1 during cell passaging under 5% O_2_ and 21% O_2_. A total of 1 × 10^5^ cells (passage 11) from the same well under 5% O_2_ were used for haploidy analysis for each group under 5% O_2_ or normoxia. Experiments were repeated three times independently, with similar results. **e** DNA FISH of chromosome X analysis of haploid cells in hAGHESC-1. Same number of cells at passage 12 from one well were used for culture under 5% and 21% O_2_, respectively. DNA FISH was performed at passage 15 and passage 20. Two-tailed student’s *t*-test. ****P < 0.0001; *P < 0.05. **f** Histological image of teratoma section derived from hAGHESC-2 (left, scale bar, 100 μm). Endoderm (respiratory epithelium), mesoderm (cartilage) and ectoderm (pigmentary epithelium) are shown in the left panel. Red box is magnified in Middle (scale bar, 20 μm) and Right (DNA FISH image, scale bar, 20 μm). *, haploid nucleus; ▲, diploid nucleus. ChrX, green. **g** DNA methylation profiles of known imprinting control regions in human sperm- and oocyte-originated haESCs using WGBS analysis. hAG-1 and hAG-2 are two sperm-originated haESC lines derived in this study. hPG-1 and hPG-2 are two oocyte-originated haESCs derived in our previous study.^2^ HUES42 is a diploid ESC line derived from ICSI blastocysts in this study. ICA-1 and ICA-2 are two diploid ESC lines derived from ICAHCI blastocysts (see Fig. 1i and Supplementary information, Fig. S5). Blood is a somatic control collected and sequenced in this study. Six somatic samples from column 12 to column 17 are from previously reported data.^15^ **h** DNA methylation analysis of the DMRs of *H19*, *IG* and *SNRPN* in hAGHESC-2 cells (passages 5 and 75), hPGHESC-2 cells (passage 29), human sperms, and fibroblasts. Filled and open circles represent methylated and unmethylated CpG sites, respectively. **i** Schematic diagram of generation of reconstructed embryos with hAG-haESCs (termed ICAHCI embryos), followed with characterization of resulting blastocysts. ASL, androgenetic spindle-like structure; MPN, maternal pronucleus; APN, androgenetic pronucleus; PPB, pseudopolar body; PB2, second polar body; TE, trophectoderm; and ICM,  inner cell mass. **j** The representative spindle-viewer image of reconstructed embryos after fusion between MII oocytes and hAG-haESCs. Two spindle-like structures (ASL and CSC) in the red box in the left image is magnified in the right image. **k** Developmental efficiency of the ICAHCI and control ICSI embryos. **l**–**n** *t*-distributed stochastic neighbor embedding (*t*-SNE) based on protein-coding genes showing unbiased clustering results of single cells in the ICAHCI and ICSI blastocysts. Cells are colored based on cell types (**l**), experiment groups (**m**) and embryos (**n**), respectively. ICSI_E8a/b in **n** represent two ICSI embryos generated with the oocytes and sperm from the same pair of donors. EPI, Epiblast; and PE, primitive endoderm. **o** The numbers of differentially expressed genes (DEGs) in TE and EPI between ICAHCI and ICSI embryos. The threshold of DEGs is 4-fold change. Violet, highly expressed in ICSI groups; yellow, highly expressed in ICAHCI groups. **p** Methylation analysis of the DMRs of *H19*, *IG* and *SNRPN* in ICA-2 cells (passage 10).

Previous studies have shown that hypoxia is critical for stem cell maintenance and promotes the generation of induced pluripotent stem cells. Moreover, human ESCs are derived from embryonic epiblasts that reside in a physiologically hypoxic environment.^9^ We thus tested whether low oxygen concentration (5%) may promote the haploid stability and enhance the generation of haploid ESCs. Interestingly, 5% O_2_ was beneficial for haploidy maintenance of human PG-haESCs (hPG-haESCs),^2^ while slightly decreased the proliferation rate of haESCs (Supplementary information, Fig. S1c, d). Meanwhile, 5% oxygen enhanced the generation of mouse haESCs although impaired the overall efficiency of ESC derivation (Supplementary information, Fig. S1e). Finally, we examined whether 5% oxygen can help to establish haESCs and derived 5 ESC lines from a total of 11 human AG blastocysts. Strikingly, two lines contained 9.0% and 2.2% of haploid cells respectively in the initial FACS-enrichment (Fig. 1b; Supplementary information, Fig. S1f). After multiple rounds of FACS-enrichment of haploid cells, two hAG-haESC lines were successfully generated (referred to as hAGHESC-1 and hAGHESC-2) (Fig. 1c; Supplementary information, Fig. S1g, l).

hAGHESC1 and hAGHESC2 cells were maintained in primed human ESC culture medium under 5% O_2_ for over 75 passages and haploid genome integrity was stably maintained through FACS-enrichment of haploid cells every 20 passages (Supplementary information, Fig. S1h). Consistently, 5% oxygen condition promoted haploidy maintenance of hAG-haESC lines (Fig. 1d, e). Cell proliferation analysis showed that 5% oxygen indeed slightly impaired the cell growth rate, but did not alter the cell phase distributions (Supplementary information, Fig. S1i, j). Interestingly, 5% oxygen reduced the number of cells that entered S phase (Supplementary information, Fig. S1k), suggesting that low oxygen tension overall decelerates cell proliferation. hAGHESC-1 and hAGHESC-2 cells were inherited from the corresponding sperm donors, sustained pluripotency and displayed differentiation potential in vitro and in vivo (Supplementary information, Fig. S2). DNA fluorescence in situ hybridization (FISH) using human chromosome X probe confirmed the existence of haploid nuclei in cultured ESCs and differentiated cells (Fig. 1f; Supplementary information, Fig. S2), indicating that haploidy is stably maintained in both undifferentiated and differentiated human haESCs.

As hAG-haESCs carried genome from sperm, we next examined whether paternal imprints are maintained in hAG-haESCs. RNA-sequencing (RNA-seq) of hAG-haESCs and hPG-haESCs with genome from oocytes^2^ showed that paternally expressed genes were upregulated while maternally expressed genes were downregulated in hAG-haESCs; in contrast, hPG-haESCs exhibited the opposite pattern (Supplementary information, Fig. S3a, b). To assess epigenetic inheritance, we performed whole genome bisulfite sequencing (WGBS) and found that whereas the differential methylated regions (DMRs) of paternal imprints retained hypermethylation in hAG-haESCs but hypomethylation in hPG-haESCs, maternal DMRs showed completely opposite methylation patterns in hAG-haESCs and hPG-haESCs (Fig. 1g; Supplementary information, Fig. S3c). We further performed bisulfite sequencing of two paternally imprinted regions, *H19*-DMR and *IG*-DMR, and one maternally imprinted region, *SNRPN*-DMR, in hAG-haESCs at different passages and confirmed that both cell lines stably sustained hypermethylation at DMRs of *H19* and *IG* (Fig. 1h; Supplementary information, Fig. S4a). Strikingly, this hypermethylation state was stably sustained in hAGHESC2 up to passage 75 (Fig. 1h). Moreover, hAG-haESC-originated embryoid bodies (EBs) and teratomas exhibited typical paternal imprinted state (Supplementary information, Fig. S4b). Taken together, these results indicate that, different from mouse haESCs whose methylation are gradually lost during cell passaging,^10^ human oocyte^2^ or sperm-originated haESCs can stably maintain the parental imprints, probably due to the use of the primed culture conditions for human ESC derivation and passaging, which is favorable to genetic and epigenetic stability of ESCs.^11,12^

Previous studies have shown that mouse AG-haESCs with *H19* and *IG* DMR deletions mimicking the paternal imprinting state of *H19* and *Gtl2* can be used as the sperm replacement to efficiently support embryonic development,^13,14^ we next tested whether hAG-haESCs with stable imprints could also be employed to ‘fertilize’ oocytes to support pre-implantation embryonic development. To this end, we adopted a modified human nuclear transfer protocol^8^ (Fig. 1i), in which, the donor cells synchronized at metaphase were fused with oocytes to produce reconstructed embryos that were then electrically activated. Subsequently, the hAG-haESC-derived spindle-like (ASL) structure and chromosome-spindle complex (CSC) could be observed in the reconstructed embryos (Fig. 1j). In 3–4 h, the maternal pronucleus (MPN) and androgenetic pronucleus (APN) could be efficiently formed in the reconstructed embryos (110 of 130) after exclusion of the second polar body (PB2) and the pseudopolar body (PPB), respectively, resulting in ‘fertilized’ embryos containing diploid genome (Fig. 1i; Supplementary information, Table S1). These reconstructed embryos (termed ICAHCI embryos) developed to blastocyst stage at a rate of 9.1%, lower than that in control intracytoplasmic sperm injection (ICSI) experiments (21.4%) (Fig. 1k).

A total of 10 high-quality ICAHCI blastocysts (4BC or better by Gardner’s criteria) were produced from hAGHESC-1 and hAGHESC-2 cells (Fig. 1i; Supplementary information, Table S2). Preimplantation genetic screening (PGS) analysis showed that 5 of them were euploid, with the rate comparable to that of ICSI-derived blastocysts (Supplementary information, Table S3), thus excluding the possibility that the process of injection of haploid cell *per se* leads to aneuploidy of the reconstructed embryos. The single-cell RNA sequencing (scRNA-seq) analysis revealed three cell lineages in all tested ICAHCI and ICSI blastocysts based on their expression patterns of protein-coding genes and long non-coding RNA (lncRNA), i.e., epiblast (EPI), primitive endoderm (PE) and trophectoderm (TE) (Fig. 1l–n; Supplementary information, Fig. S5a-f). Interestingly, EPI and PE cells of ICAHCI and ICSI blastocysts clustered together, while TE cells of each blastocyst tended to cluster separately and exhibited heterogeneity even in the same group, reflecting that TE would be vulnerable to be affected during in vitro fertilization by sperm or hAG-haESCs. Moreover, small numbers of differentially expressed genes (DEGs) existed in EPI or TE cells between ICSI and ICAHCI embryos (Fig. 1o), which could not be enriched to any known pathways. Finally, we derived two diploid ESC lines (termed ICA1 and ICA2) from four ICAHCI blastocysts and found that both lines were originated from donor haploid cells, sustained genome integrity and exhibited pluripotency both in vitro and in vivo (Fig. S5g-j; Supplementary information, Table S2). Importantly, ICA1 and ICA2 and their teratomas showed normal DNA methylation state at three imprinted genes (Fig. 1p; Supplementary information, Fig. S5k). This indicates that imprints in hAG-haESCs are stably maintained during pre-implantation embryonic development after injection into oocytes, the same as those in sperms, and during ESC derivation from ICAHCI blastocysts, and long-term ESC passaging and differentiation. Moreover, WGBS and RNA-seq showed that ICA1 and ICA2 exhibited similar DNA methylome and transcriptome features compared to normal zygote-derived ESCs (Fig. 1g; Supplementary information, Figs. S3b, c). Taken together, these results indicate that ICAHCI embryos are generally very similar to ICSI embryos, providing a unique system for the study of human early embryonic development in vitro.

We have demonstrated that human haploid ESCs can be generated through sperm nuclear transfer under a culture condition with low oxygen concentration. hAG-haESCs containing sperm genome stably maintain typical imprinted DMRs during ESC derivation and proliferation in vitro. Strikingly, hAG-haESCs can ‘fertilize’ oocyte and support early embryonic development, leading to blastocysts and diploid ESCs with comparable transcriptome to those of normal diploid embryos and ESCs, respectively. While future study will be needed to optimize the procedure of embryo reconstruction using haploid cells and to improve their pre-implantation development, we believe that our method will provide a novel tool for facilitating genetic analysis of early human embryonic development through complex gene modifications in reconstructed embryos via haploid ESC as mediator, which enables precise analyses through preselection of haploid cells with expected genetic traits and without off-targets.

## Supplementary information

Supplementary information, Data S1

Supplementary information, figures

Supplementary information,  tables
