# Supplementary material for: In vitro expansion of human sperm through nuclear transfer
Source: Cell Res. 2019 Dec 18;30(4):356–9. doi: 10.1038/s41422-019-0265-1 (PMC7118075; doi:10.1038/s41422-019-0265-1)
Supplement: Supplementary file 1 — Supplementary information, Data S1 [file 41422_2019_265_MOESM1_ESM.pdf]

Supplementary information, Data S1. Materials and methods

Source of human germ cells and other tissues

This study was approved by the Institutional Review Board of Reproductive Medicine of Shandong University, and informed consent was signed by all participants. The human sperm samples were redundant sperms collected after the regular therapies. Oocytes (453 in total) used in this study were all immature oocytes in metaphase-I (MI) or germinal vesicle (GV) phase donated by patients for IVF or ICSI treatment. Follicular fluid samples were collected from female donors in the process of hormone induction ovulation. The remaining sperm after the IVF was collected for ICSI or extracting the genome.

***In vitro* maturation (IVM) of human oocytes**

The oocyte corona-cumulus complexes (OOCC) were cultured with G-IVF medium (Vitrolife) for two to five hours (37 °C, 6% CO_2). Then, OOCCs were digested with hyaluronidase (Sigma) to remove the cumulus cells. Next, MI and GV stage immature oocytes were picked out and *in vitro* matured to metaphase-II (MII) stage using IVM culture medium for 24 to 28 hours. The IVM oocytes with the first polar body extrusion were used in this study. IVM culture medium was composed of the following reagents, including TCM-199, 10% HSA, 50 U/ml penicillin, 50 U/ml streptomycin, 10 ng/ml EGF, 0.075 U/ml FSH and 0.15 U/ml HCG.

Construction of human androgenetic haploid embryo

The IVM oocytes were fertilized by ICSI and activated by the human sperm. The zygotes were cultured (5% CO_2, 5% O_2, 37 °C) for another two hours, and then the maternal chromatin was removed together with the pre-pronucleus (PPN) [1], resulting in embryos carrying only sperm genome (termed human androgenic haploid embryos). Androgenic embryos were then cultured with G1 medium (5% CO_2, 5% O_2, 37 °C) to eight-cell stage and then changed to G2 medium for further culturing (5% CO_2, 5% O_2, 37 °C) to blastocyst stage. All high-quality blastocysts were frozen for further analysis.

Derivation of human androgenetic haploid embryonic stem cell (hAG-haESC) lines

The human androgenic blastocysts were thawed followed the standard procedure and the trophectodermal cells were ablated by laser system. Next, the inner cell mass (ICM) were planted on mitotically inactivated mouse embryonic fibroblasts (MEFs) in modified human embryonic stem cell culture medium, composed with Knock-out DMEM (Gibco), 20% KSR (Gibco), 0.1 mM nonessential amino acids (Invitrogen), 2 mM L-glutamine, 0.1 mM β-mercaptoethanol, 50 U/ml penicillin, 50 μg/ml streptomycin, 30 ng/ml bFGF (Gibco), 2 μM Thiazovivin (Selleck) and 10 μM Y-27632 (Selleck). ICMs were usually attached to the feeders in three days, followed by further expansion with culture medium change every two days. Outgrowths were formed after seven days and passaged on fresh MEF feeders followed by mechanically separating into several pieces. After two rounds of mechanical passage, the cultured ESCs reached the required cell number (>100 thousand) for FACS-enrichment of haploid cells. The primed human ESC culture medium was maintained in a humidified incubator at 37°C, 5% O_2 or 21% O_2.

FACS-enrichment of haploid cells

Haploid ESCs need to be enriched regularly during the process of passaging. Briefly, human ESCs were dissociated into single cells with Accutase (Gibco) and then incubated with 15 μg/ml Hoechst-33342 at 37 °C for 15 min, followed by centrifugation (100 g) and resuspension with human ESC culture medium with 10 μM ROCK inhibitor Y-27632. Next, the stained cells were filtered with 40 μm strainers and the haploid cells were sorted on BD FACS Aria II (BD Biosciences) with the 355 nm laser. Usually, diploid ESCs were used as negative control and oocyte-originated haploid ESCs were used as positive control for sorting hAG-haESCs. After enrichment, the haploid cells were seeded on fresh MEF feeders in ESC culture medium supplied with 10 μM ROCK inhibitor Y-27632. ESC clones were formed in five days and ESCs were then passaged using single cell passage method through digestion by Accutase.

Karyotype analysis

Human ESCs were passaged to three wells of a 6-well plate, each with 500-thousand cells. After two days of culturing, the cells were incubated with 140 ng/ml demecolcine (Sigma) for 8 hours. Then, the cells were digested into single cells with Accutase and centrifuged (100 g) to remove supernatant. Next, the cell pellets were resuspended with 0.075 M KCl at 37 °C for 30 min. Hypotonic solution-treated cells were pre-fixed with methanol: acetic acid (3:1 in volume) followed by centrifugation (400 g) and then fixed again in methanol: acetic acid (3:1 in volume) on ice for another 2 hours. Finally, the fixed cells were dropped on ice-cold clean slides followed by metaphase spread and stained with Giemsa before photographing. Each chromosome imagine was arranged based on the Giemsa-binding pattern.

Genome integrity analysis

Genome integrity analysis was processed with VeriSeq PGS Kit (Illumina). Briefly, one to three trophectoderm cells of blastocysts were biopsied, collected into PCR tubes containing 2.5 μL PBS. The quality of DNA samples was analyzed using Qubit 2.0 Fluorometer (Invitrogen). For library preparing, one nanogram DNA was fragmented and labeled with transposase, Tn5, and then amplified by PCR. The libraries were sequenced on the Miseq system (Illumina) and analyzed with BlueFuse Multi Analysis Software (Illumina).

Short tandem repeat (STR) analysis

STR analysis was used in the paternity test with PowerPlex® 21 System. Specifically, for bulk cell samples including human ESCs and blood samples, genomic DNA was extracted and quality of DNA was checked with NanoDrop (ThermoFisher). The extracted DNA was amplified with the following thermal cycling protocol, 96 °C for 1 minute then 94 °C for 10 seconds 59 °C for 1 minute 72 °C for 30 seconds for 30 cycles, next 60 °C for 10 minutes, finally 4 °C hold forever. After amplification, the samples proceeded with fragments analysis using the genetic analyzer (Applied Biosystems® 3500). Data analysis was processed with GeneMapper® ID-X Software (version: 1.2).

For single-cell scale STR analysis, the biopsied TE cells were stored in PCR tube containing 3 μL PBS, and then the genome was amplified with VeriSeq PGS Kit (Illumina). The amplified genome DNA was then performed analysis as extracted DNA mentioned above.

Reconstructed embryos from hAG-haESCs

Cultured hAG-haESCs were incubated with 70 ng/ml demecolcine (Sigma) for 12 hours to block the cell cycle into metaphase followed by dissociated into single cells by Accutase. Then, the suspension was centrifuged (100 g) and the cell pellet was resuspended in 500 μl G1 medium supplied with 10 μM ROCK inhibitor Y-27632. High-quality metaphase ha-AGHESCs with smooth surface were picked and fused with oocytes, at the position against the second polar body, mediated by Sendai virus (COSMO BIO CO., LTD) in a standard culture environment (5% CO_2, 5% O_2, 37 °C), and at this time we termed this kind of embryos as ICAHCI human embryos. After 30 minutes incubation, the ICAHCI human embryos were activated followed the procedure previously reported [2], briefly, with two electroporation pulses (50 ms DC pulses of 2.7 kV cm-1) (Model PA-400, Cyto Pulse Science) in the d-sorbitol buffer. Activated human ICAHCI-embryos were then cultured in G1 medium (5% CO_2, 5% O_2, 37 °C) and two pro-pronuclei could be figured out after 10 hours. Eight-cell stage ICAHCI -embryos were transferred into the G2 culture medium (5% CO_2, 5% O_2, 37 °C) to obtain blastocysts. ICAHCI blastocysts were frozen after biopsied with the laser to collect one to two TE cells used in genome integrity analysis.

Embryoid body (EB) formation analysis

The cultured embryonic stem cell clones were digested with collagenase IV in 37 °C for 20 minutes followed by centrifugation (100 g, 5 min) to remove the supernatant. Secondly, the cell pellets were washed with PBS. Next, the cell pellets were resuspended in EB culture medium (DMEM/F12, 15% FBS, 2 mM L-glutamine Invitrogen, 0.1 mM nonessential amino acids, 0.1 mM β-mercaptoethanol, 50 U/ml penicillin and 50 μg/ml streptomycin) for five days (37 °C, 5% CO_2). The EBs were

pipetted out, dissociated with TrypLE Express (Gibco) and re-plated on matrigel-coated slides for additional 3 days before Immunofluorescence staining.

Teratoma formation analysis

For teratoma formation, around five million cultured embryonic stem cells were harvested with collagenase IV and washed with MEF culture medium (DMEM, 10% FBS, 0.1 mM nonessential amino acids, 50 U/ml penicillin and 50 μg/ml streptomycin). The cell pellets were resuspended with 120 ul injection medium, a mixture of hESC culture medium and matrigel with equal volume and supplied with 10 μM Y-27632. The resuspension was intramuscularly or subcutaneously injected to SCID/NOD mice using insulin syringe (BD). Teratomas were harvested and dissected into several pieces for further analysis. For histochemical stain, the teratomas were fixed in 4% paraformaldehyde in 4 °C. For cell culture, however, the teratomas were digested with 0.05% trypsin and cultured with MEF culture medium.

Immunofluorescence staining

For immunofluorescence staining, cells grown on glass slides, pre-coated with Matrigel (Corning), was fixed with 4% paraformaldehyde for 15 minutes at room temperature, next permeabilized for 15 minutes in 0.2% Triton-X100 in PBS and then blocked using 1% BSA in PBS. Human ESC clones were incubated with antibodies, including OCT4 (POU5F1) (SantaCruz, sc-9081, 1:100), SOX2 (Millipore, AB5603, 1:100), SSEA4 (SantaCruz, sc-21704, 1:100), TRA-1-60 (Santa Cruz, sc-21705, 1:100), TRA-1-81 (Santa Cruz, sc-21706, 1:100), Ki-67 (Cell Signaling Technology, #9449, 1:800) and Phospho-Histone H3 (Ser10) (Cell Signaling Technology, #53348, 1:1600), overnight in 4 °C or 2 hours at room temperature. Differentiated cells were incubated with three germ-layers makers, including α-TUJ (R&D, MAB1195, 1:1000) for ectoderm, α-SMA (Abcam, ab5694, 1:1000) for mesoderm and AFP (R&D, MAB1368, 8 μg/mL) for endoderm. Then the slides were washed with PBS containing 0.05% Tween-20 (Thermofisher) for three times in room temperature, each time for 5 minutes, and then incubated with fluorescently coupled secondary antibody for 1 hour in room

temperature. All the antibodies were diluted with blocking buffer. Finally, the slides were washed and mounted before photography.

BrdU pulse-labeling

Human ESCs were plated into the cell culture well with glass slide (8mm * 8mm) that pretreated with mouse fibroblast feeder cells. After two days while hESCs formed clones, fresh warmed hESC culture medium with BrdU labeling reagent (Thermofisher, # 000103, 1:100) was added into the wells for 1 hour. After treatment, the slides were washed with warmed PBS and fixed with 4% PFA (in PBS) for 20 min. The following treatment was similar as previous mentioned step in Immunofluorescence staining.

Bisulfite PCR

Four hundred nanograms extracted DNAs were performed bisulfite conversion using EZ DNA methylation Gold kit (ZYMO Research). The PCR products were cloned to PMD-19T vectors (Takara) and single colonies were sequenced with primer M13F. Bisulfite-PCR primers were designed with Methyl Primer Express Software (version: 1.0, ABI) while primers target H19-DMR, IG-DMR and SNRPN-DMR were from the previous report [3].

Single-cell RNA-seq

Single cells were picked into the lysis buffer using a mouth pipette after washing in BSA for several times. And then single-cell RNA-seq libraries were generated following a modified STRT-seq protocol as previously described [4-6]. In brief, an oligo-dT primer anchored with an 8 nt barcode and unique molecular identifiers (UMI) was used to capture mRNAs from cells and reverse transcribed for the first-strand cDNAs. Second-strand cDNAs were synthesised by 18 cycles of PCR amplification. Then the amplified cDNAs with different barcodes were pooled together followed by 4 cycles' PCR using biotinylated pre-indexed primers. After DNA fragmentation by Covaris S2 instrument (Covaris), the 3' cDNAs with biotin modification were captured through the use of Dynabeads MyOne Streptavidin C1 beads (Thermo Fisher Scientific). Finally,

the libraries were constructed using a Kapa Hyper Prep Kit (Kapa Biosystems) and sequenced by Illumina HiSeq 4000 platform with a 150-bp paired-end read strategy (Novogene).

Whole-genome bisulfite sequencing (WGBS)

Genomic DNA was extracted from cell lines using the DNeasy Blood & Tissue Kit (Qiagen). There were two repetitions per cell line. For WGBS library construction, the DNA was fragmented into 300 bp by sonication using Covaris S2 instrument (Covaris), following end repair and A-tailing. Cytosine methylated adaptor-ligated DNA was treated with sodium bisulfite through an EZ-96 DNA Methylation-Direct™ MagPrep (Zymo Research). Next, we used KAPA HiFi HotStart Uracil+ ReadyMix (Kapa Biosystems) to accomplish 4 cycles of PCR amplification. It should be noted that DNA was treated with sodium bisulfite twice to reach >99% bisulfite conversion rate and lambda DNA was added to assess the rate.

RNA extraction and sequencing

Total RNA was extracted from cell lines used in the WGBS study through a RNeasy Mini Kit (Qiagen) following the manufacturer's instructions. Residual DNA was removed by RNase-free DNase Set (Qiagen). For rRNA-depleted RNA-seq library preparation, total RNA was treated by a NEBNext® rRNA Depletion Kit (NEB). The cDNAs were synthesized by a NEBNext® RNA First-Strand Synthesis Module (NEB) and the second strand was synthesized by a NEBNext® Ultra II Non-Directional RNA Second Strand Synthesis Module (NEB). Final libraries were prepared using a Kapa Hyper Prep Kit (Kapa Biosystems) once again. Both WGBS and bulk RNA-seq libraries were sequenced on the HiSeq 4000 (Novogene).

Processing single-cell RNA-seq

Raw single-cell RNA-seq data were firstly trimmed the template switch oligo and polyA sequence using customer scripts, and then reads with adapters or low-quality bases were removed. Clean reads were then aligned into the human reference genome

(hg19) using *Tophat* (version: 2.0.12) [7], and *HTSeq* [8] was used to count the unique UMI molecules based on the Ensembl hg19 annotation reference, that is to say, we only quantified the UMI once and removed the duplicated UMI.

We quantified the gene *g* expression level in the single cell *c* as the transcripts per million (TPM), the UMIs number of the gene *g* dividing by the sum of UMIs number in the cell *c*, then multiplying by 100,000. We transformed the gene expression level into $\log_2$(TPM + 1).

To obtain cells with high quality, we filtered cells with three criterions: the rate of reads mapped against the reference genome greater than 40%, the number of genes detected in the Ensembl annotation reference greater than 2,000, and the number of UMIs greater than 10,000. After the strict filtering process, there were 745 of 861 single cells left for the downstream analysis.

Identification of cell types according to single-cell RNA-seq data

For single-cell RNA-seq, we identified cell types based on protein-coding genes or long non-coding RNAs (lncRNAs) with the R package *Seurat* (version: 2.3.4) [9]. In the Ensembl annotation reference, there are 577,773 annotation items including 20,327 protein-coding genes and 13,346 lncRNAs (consisting of 'lincRNA', 'antisense', 'sense_overlapping', 'sense_intronic' and '3prime_overlapping_ncrna' in annotation items). Only genes or lncRNAs expressed in at least 3 cells were left for this analysis. We firstly selected 833 highly variable genes or 24 highly variable lncRNAs using the function *FindVariableGenes* with parameters '*x.low.cutoff = 1, x.high.cutoff = 10, y.cutoff = 1*'. Based on these genes or lncRNAs, we performed the principal component analysis (PCA) using the function *RunPCA*, and then selected the significant PC dimensions (PC1~PC18 for protein-coding genes, PC1~PC4 for lncRNAs) for the *t*-SNE analysis. For identifying cell types, we then classified single cells into several clusters using the function *FindClusters* with the special clustering parameter '*resolution*' (10 clusters with '*resolution*' = 1 for protein-coding genes, 16 clusters with '*resolution*' = 3 for lncRNAs). According to well-known cell type specific marker genes (for example, *NANOG* is the EPI marker, *GATA4* for PE and *GATA2* for TE), we merged

some clusters and defined cell types.

Processing whole-genome bisulfite sequencing (WGBS) data

Adaptors and low-quality bases were removed from raw WGBS data using *trim_galore* (version: 0.1.3) with parameters '*--quality 20 --stringency 3 --length 50 --clip_R1 9 --clip_R2 9 --paired --trim1 --phred33*'. The clean reads were then paired mapped against the human reference genome hg19 using *Bismark* (version: 0.7.6) [10] with parameters '*--fastq --non_directional --phred33-quals*'. For improving the number of mapped reads, we re-mapped the unmapped reads with the single-end aligned mode. Only uniquely mapped reads were retained for the downstream analysis.

Processing bulk RNA-seq data

Adaptors and low-quality bases were removed from raw RNA-seq sequencing data using the custom script. The clean reads were then mapped against the Ensembl human reference genome hg19 using *TopHat* (version: 2.0.12) [7] with default parameters. Raw reads were counted with *HTSeq* [8], and the gene expression level was quantified with the RPKM (reads per kilobase million).

Principal components analysis (PCA) based on RNA-seq or DNA methylation data

We performed PCA and unsupervised clustering analysis to detect the heterogeneity of different samples. For RNA-seq or DNA methylation, we performed PCA with the R function *procomp*.

Notably, for DNA methylation analysis, we used the 1× coverage CpG methylation data. When we performed PCA and unsupervised clustering analysis in the whole genome, we calculated the mean DNA methylation level in each 1-kb tile only if more than five CpG sites were covered within this tile. We removed some tiles with null values to perform this analysis.

Collection of published somatic cell DNA methylation data

We collected DNA methylation data of somatic cells from the previous study [12]. We

also used the 1× coverage CpG methylation data to investigate methylation levels in differential methylated regions of paternal and maternal imprints.

Data accession

The raw data of scRNA-seq and processed data of scRNA-seq, bulk RNA-seq and WGBS in this study are deposited at Gene Expression Omnibus (GEO) with accession number GSE128377, and the raw data of WGBS, scRNA-seq and bulk RNA-seq are deposited at Genome Sequence Archive (GSA, https://ngdc.cncb.ac.cn/) for Human with accession number HRA004100.

Reference

1 Wu, K. *et al.* Polar bodies are efficient donors for reconstruction of human embryos for potential mitochondrial replacement therapy. *Cell Res* **27**, 1069-1072, doi:10.1038/cr.2017.67 (2017).

2 Tachibana, M. *et al.* Human embryonic stem cells derived by somatic cell nuclear transfer. *Cell* **153**, 1228-1238, doi:10.1016/j.cell.2013.05.006 (2013).

3 Zhong, C. Q. *et al.* Generation of human haploid embryonic stem cells from parthenogenetic embryos obtained by microsurgical removal of male pronucleus. *Cell Res* **26**, 743-746, doi:10.1038/cr.2016.59 (2016).

4 Cui, Y. *et al.* Single-Cell Transcriptome Analysis Maps the Developmental Track of the Human Heart. *Cell Rep* **26**, 1934-1950 e1935, doi:10.1016/j.celrep.2019.01.079 (2019).

5 Islam, S. *et al.* Characterization of the single-cell transcriptional landscape by highly multiplex RNA-seq. *Genome Res* **21**, 1160-1167, doi:10.1101/gr.110882.110 (2011).

6 Picelli, S. *et al.* Full-length RNA-seq from single cells using Smart-seq2. *Nat Protoc* **9**, 171-181, doi:10.1038/nprot.2014.006 (2014).

7 Trapnell, C., Pachter, L. & Salzberg, S. L. TopHat: discovering splice junctions with RNA-Seq. *Bioinformatics* **25**, 1105-1111 (2009).

8 Anders, S., Pyl, P. T. & Huber, W. HTSeq—a Python framework to work with

high-throughput sequencing data. *Bioinformatics* **31**, 166-169 (2015).

9 Satija, R., Farrell, J. A., Gennert, D., Schier, A. F. & Regev, A. Spatial reconstruction of single-cell gene expression data. *Nature biotechnology* **33**, 495-502 (2015).

10 Krueger, F. & Andrews, S. R. Bismark: a flexible aligner and methylation caller for Bisulfite-Seq applications. *bioinformatics* **27**, 1571-1572 (2011).

11 Guo, F. *et al.* The transcriptome and DNA methylome landscapes of human primordial germ cells. *Cell* **161**, 1437-1452 (2015).
