# Supplementary material for: In vitro expansion of human sperm through nuclear transfer
Source: Cell Res. 2019 Dec 18;30(4):356–9. doi: 10.1038/s41422-019-0265-1 (PMC7118075; doi:10.1038/s41422-019-0265-1)
Supplement: Supplementary file 2 — Supplementary information, figures [file 41422_2019_265_MOESM2_ESM.pdf]

Figure S1

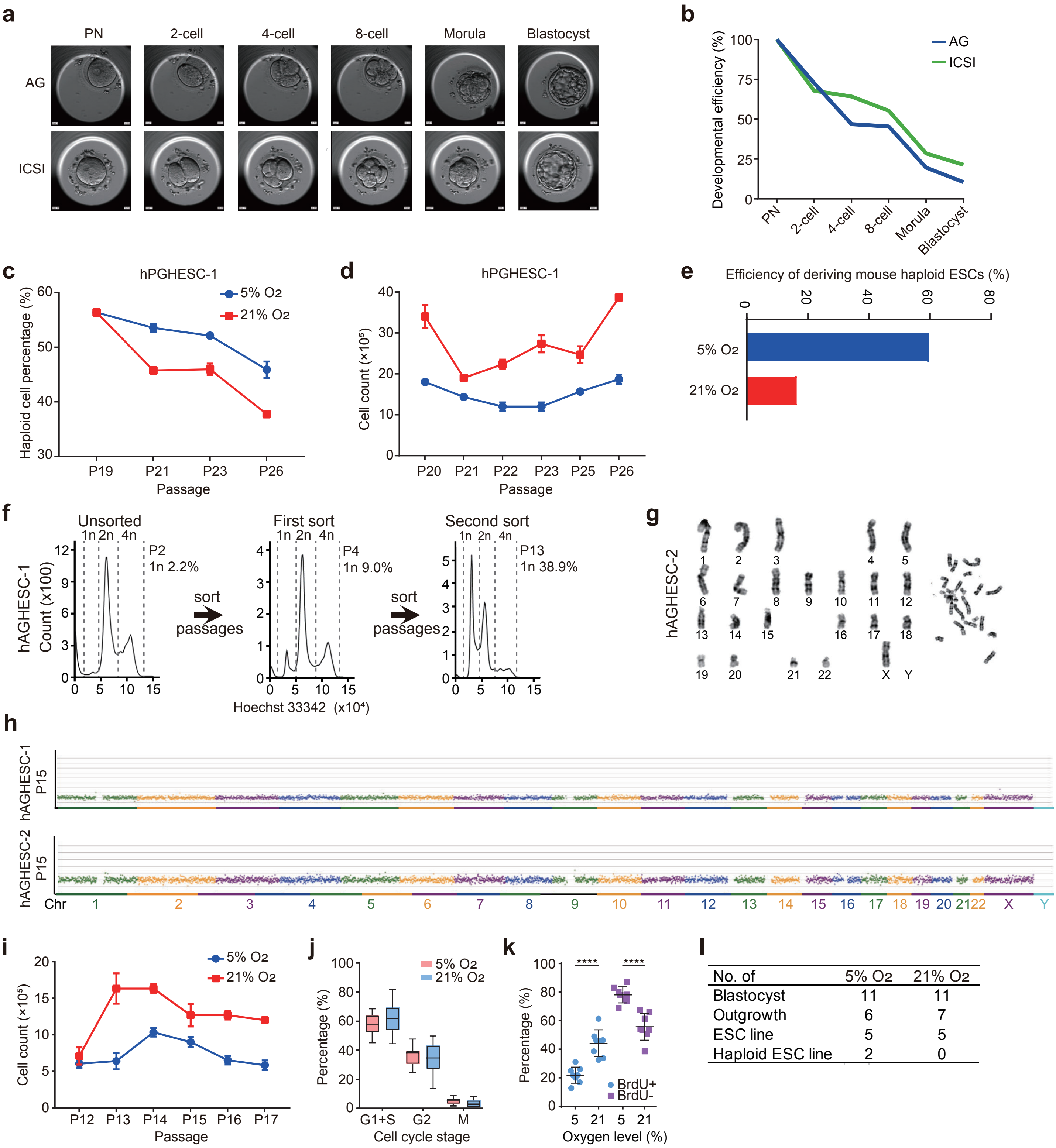

No. of	5% O2	21% O2
Blastocyst	11	11
Outgrowth	6	7
ESC line	5	5
Haploid ESC line	2	0

Figure S2

a

STR	Female #1		Male #1		hAGHESC-1	Female #2		Male #2		hAGHESC-2
D3S1358	16	16	16	17	17	18	18	15	15	15
D6S1043	13	19	14	14	14	12	19	10	18	18
D13S317	9	11	13	9	9	10	10	11	12	12
Penta E	15	16	22	16	16	12	16	11	11	11
D16S539	9	11	11	11	11	11	12	9	9	9
D18S51	13	17	23	13	13	16	17	15	14	14
D2S1338	20	24	24	20	20	23	23	19	23	23
CSF1PO	9	10	11	11	11	10	13	12	11	11
Penta D	11	13	10	11	11	12	14	13	9	9
THO1	7	9	7	9	9	7	9	9	9	9
vWA	19	19	18	14	14	14	17	17	18	18
D21S11	29	33	31.2	29	29	30	30.2	33.2	29	29
D7S820	10	11	11	11	11	11	12	12	11	11
D5S818	11	12	11	11	11	10	12	13	11	11
TPOX	8	9	8	10	10	11	11	8	11	11
D8S1179	12	14	13	11	11	10	14	13	13	13
D12S391	15	21	20	22	22	15	24	21	20	20
D19S433	13	14.2	15	13.2	13.2	13	15.2	13	14.2	14.2
FGA	21	23	19	23	23	22	23	24	20	20
	X	X	Y	X	X	X	X	Y	X	X

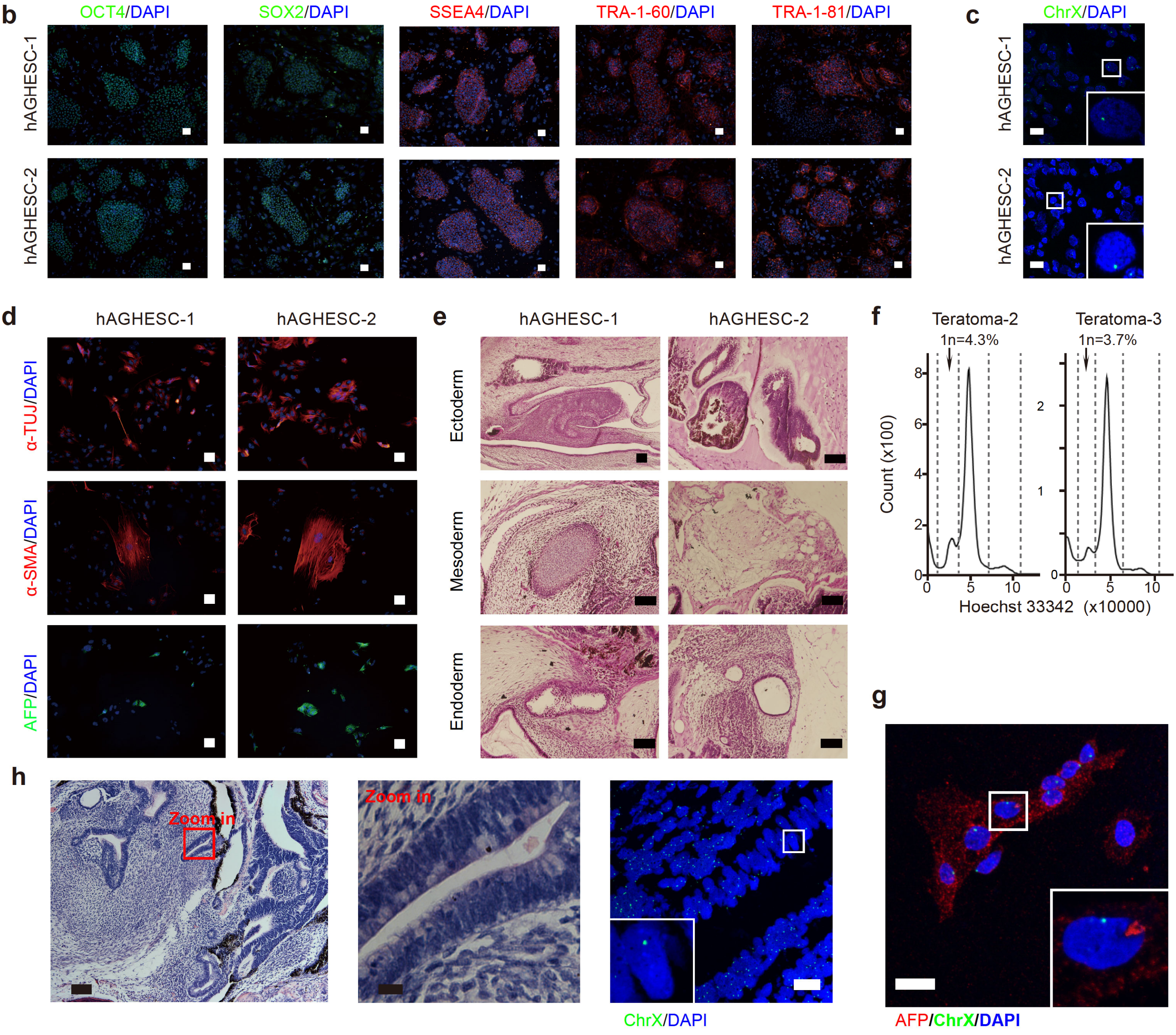

Figure S3

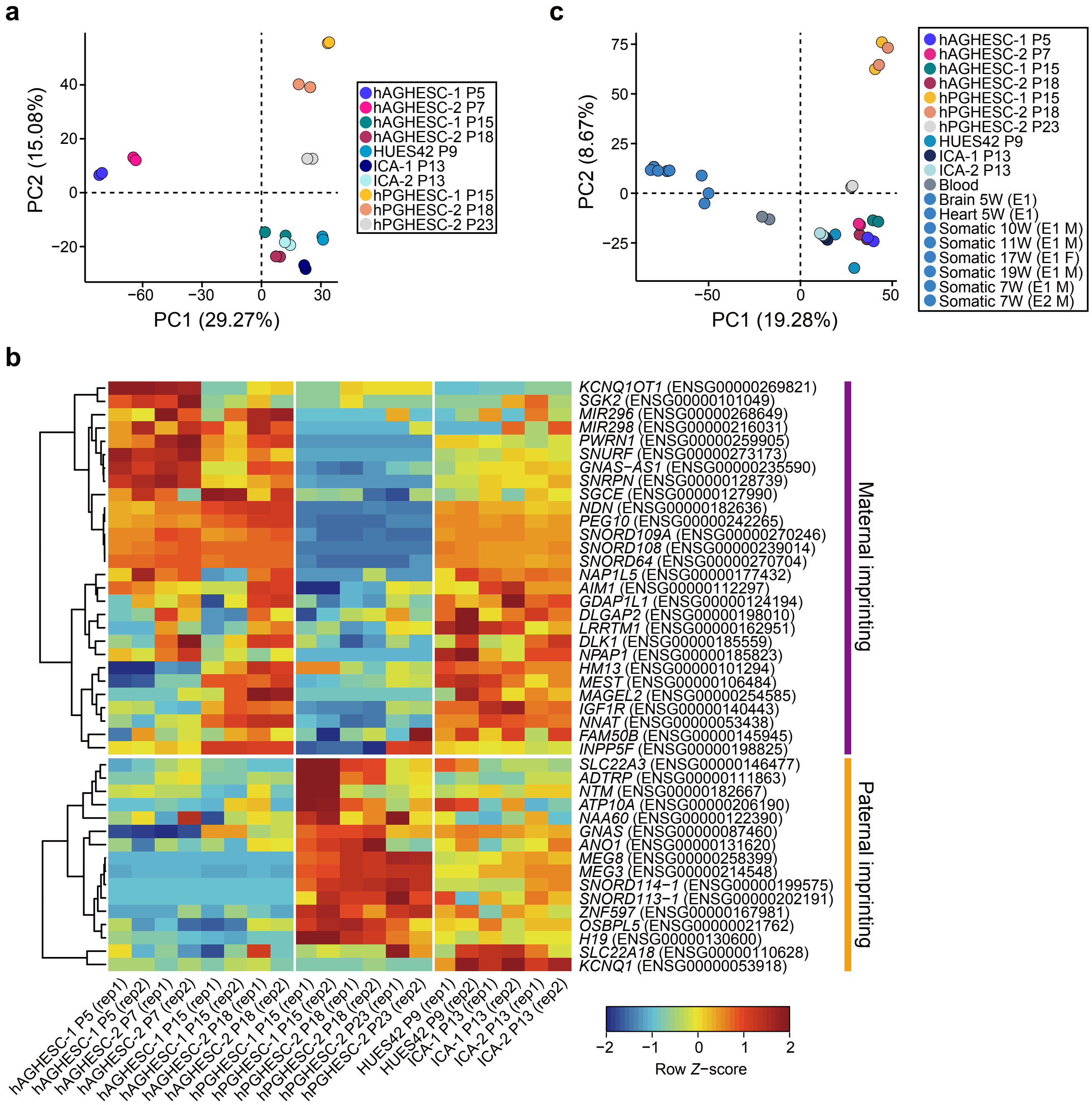
a
PC2 (15.08%)
PC1 (29.27%)
hAGHESC-1 P5
hAGHESC-2 P7
hAGHESC-1 P15
hAGHESC-2 P18
HUES42 P9
ICA-1 P13
ICA-2 P13
hPGHESC-1 P15
hPGHESC-2 P18
hPGHESC-2 P23
c
PC2 (8.67%)
PC1 (19.28%)
hAGHESC-1 P5
hAGHESC-2 P7
hAGHESC-1 P15
hAGHESC-2 P18
hPGHESC-1 P15
hPGHESC-2 P18
hPGHESC-2 P23
HUES42 P9
ICA-1 P13
ICA-2 P13
Blood
Brain 5W (E1)
Heart 5W (E1)
Somatic 10W (E1 M)
Somatic 11W (E1 M)
Somatic 17W (E1 F)
Somatic 19W (E1 M)
Somatic 7W (E1 M)
Somatic 7W (E2 M)
b
KCNQ1OT1 (ENSG00000269821)
SGK2 (ENSG00000101049)
MIR296 (ENSG00000268649)
MIR298 (ENSG00000216031)
PWRN1 (ENSG00000259905)
SNURF (ENSG00000273173)
GNAS−AS1 (ENSG00000235590)
SNRPN (ENSG00000128739)
SGCE (ENSG00000127990)
NDN (ENSG00000182636)
PEG10 (ENSG00000242265)
SNORD109A (ENSG00000270246)
SNORD108 (ENSG00000239014)
SNORD64 (ENSG00000270704)
NAP1L5 (ENSG00000177432)
AIM1 (ENSG00000112297)
GDAP1L1 (ENSG00000124194)
DLGAP2 (ENSG00000198010)
LRRTM1 (ENSG00000162951)
DLK1 (ENSG00000185559)
NPAP1 (ENSG00000185823)
HM13 (ENSG00000101294)
MEST (ENSG00000106484)
MAGEL2 (ENSG00000254585)
IGF1R (ENSG00000140443)
NNAT (ENSG00000053438)
FAM50B (ENSG00000145945)
INPP5F (ENSG00000198825)
SLC22A3 (ENSG00000146477)
ADTRP (ENSG00000111863)
NTM (ENSG00000182667)
ATP10A (ENSG00000206190)
NAA60 (ENSG00000122390)
GNAS (ENSG00000087460)
ANO1 (ENSG00000131620)
MEG8 (ENSG00000258399)
MEG3 (ENSG00000214548)
SNORD114−1 (ENSG00000199575)
SNORD113−1 (ENSG00000202191)
ZNF597 (ENSG00000167981)
OSBPL5 (ENSG00000021762)
H19 (ENSG00000130600)
SLC22A18 (ENSG00000110628)
KCNQ1 (ENSG00000053918)
Maternal imprinting
Paternal imprinting
hAGHESC-1 P5 (rep1)
hAGHESC-1 P5 (rep2)
hAGHESC-2 P7 (rep1)
hAGHESC-2 P7 (rep2)
hAGHESC-1 P15 (rep1)
hAGHESC-1 P15 (rep2)
hAGHESC-2 P18 (rep1)
hAGHESC-2 P18 (rep2)
hPGHESC-1 P15 (rep1)
hPGHESC-1 P15 (rep2)
hPGHESC-2 P18 (rep1)
hPGHESC-2 P18 (rep2)
hPGHESC-2 P23 (rep1)
hPGHESC-2 P23 (rep2)
HUES42 P9 (rep1)
HUES42 P9 (rep2)
ICA-1 P13 (rep1)
ICA-1 P13 (rep2)
ICA-2 P13 (rep1)
ICA-2 P13 (rep2)
Row Z−score

Figure S4

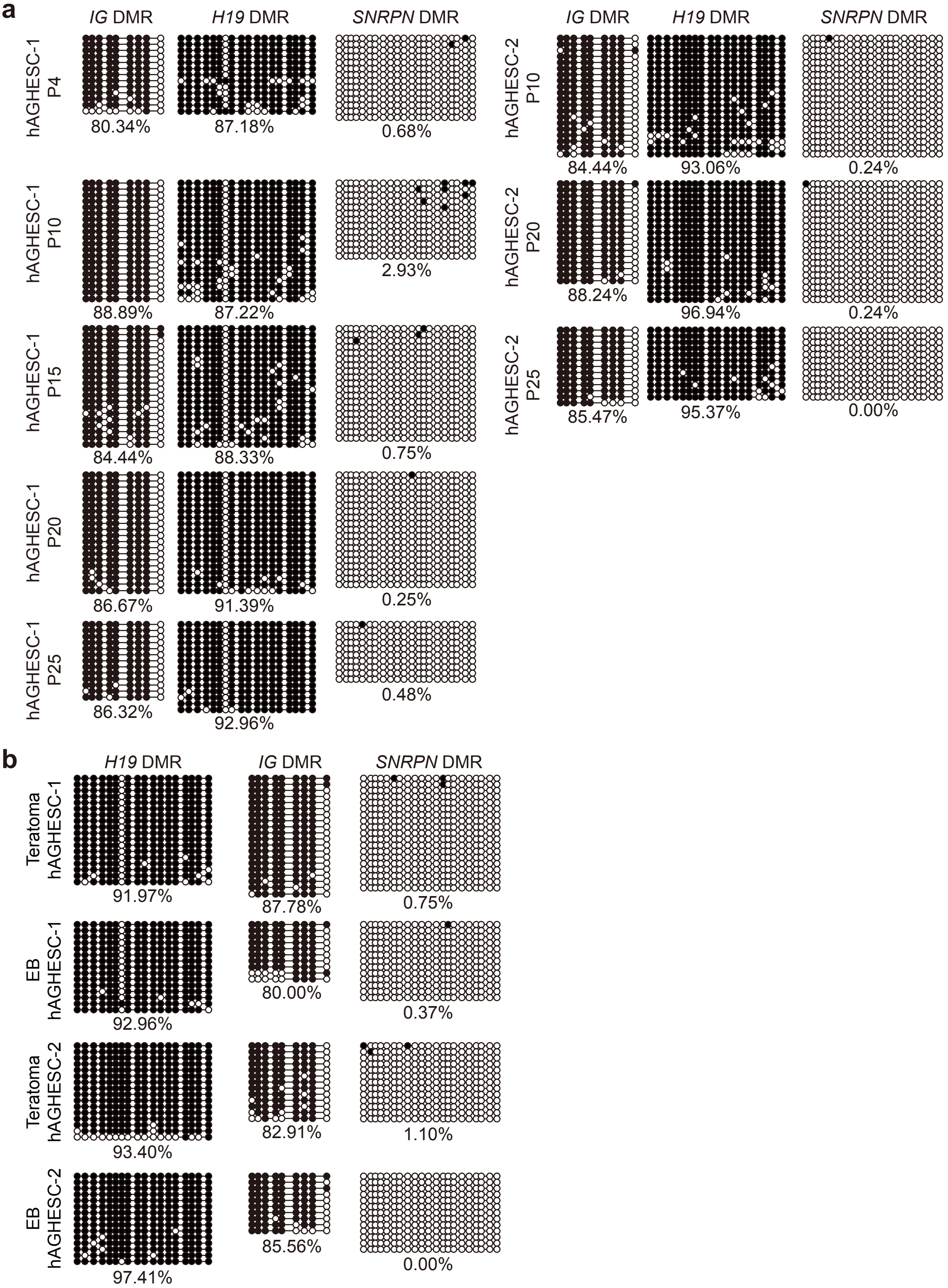

a
IG DMR
H19 DMR
SNRPN DMR
hAGHESC-1 P4
80.34%
87.18%
0.68%
hAGHESC-1 P10
88.89%
87.22%
2.93%
hAGHESC-1 P15
84.44%
88.33%
0.75%
hAGHESC-1 P20
86.67%
91.39%
0.25%
hAGHESC-1 P25
86.32%
92.96%
0.48%
IG DMR
H19 DMR
SNRPN DMR
hAGHESC-2 P10
84.44%
93.06%
0.24%
hAGHESC-2 P20
88.24%
96.94%
0.24%
hAGHESC-2 P25
85.47%
95.37%
0.00%
b
H19 DMR
IG DMR
SNRPN DMR
Teratoma hAGHESC-1
91.97%
87.78%
0.75%
EB hAGHESC-1
92.96%
80.00%
0.37%
Teratoma hAGHESC-2
93.40%
82.91%
1.10%
EB hAGHESC-2
97.41%
85.56%
0.00%

Figure S5

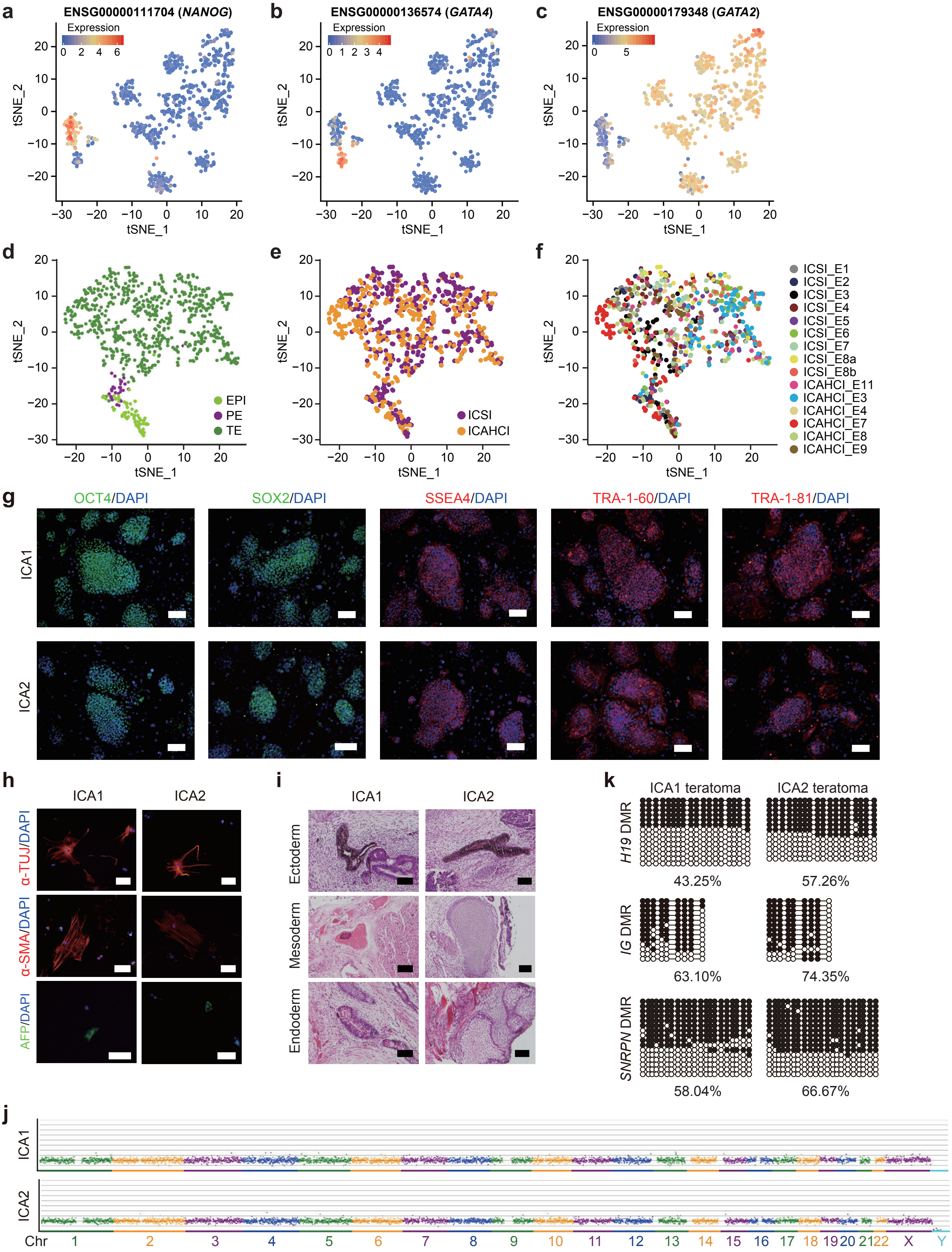
a
ENSG00000111704 (NANOG)
b
ENSG00000136574 (GATA4)
c
ENSG00000179348 (GATA2)
Expression
tSNE_1
tSNE_2
d
e
f
EPI
PE
TE
ICSI
ICAHCI
ICSI_E1
ICSI_E2
ICSI_E3
ICSI_E4
ICSI_E5
ICSI_E6
ICSI_E7
ICSI_E8a
ICSI_E8b
ICAHCI_E11
ICAHCI_E3
ICAHCI_E4
ICAHCI_E7
ICAHCI_E8
ICAHCI_E9
g
OCT4/DAPI
SOX2/DAPI
SSEA4/DAPI
TRA-1-60/DAPI
TRA-1-81/DAPI
ICA1
ICA2
h
α-TUJ/DAPI
α-SMA/DAPI
AFP/DAPI
i
Ectoderm
Mesoderm
Endoderm
k
ICA1 teratoma
ICA2 teratoma
H19 DMR
43.25%
57.26%
IG DMR
63.10%
74.35%
SNRPN DMR
58.04%
66.67%
j
Chr
1 2 3 4 5 6 7 8 9 10 11 12 13 14 15 16 17 18 19 20 21 22 X Y

Supplementary information, Figures.

Figure S1. Derivation of hAG-haESCs.

(a) Development of AG and ICSI embryos *in vitro*.

(b) Developmental efficiency of androgenetic (AG) haploid embryos and ICSI embryos.

(c) Percentages of haploid cells in hPGHESC-1 cells during cell passaging under 5% O_2 or 21% O_2. A total of 1×10^5 cells (passage 19) from the same well under 5% oxygen were used for haploidy analysis for each group under 5% O_2 or 21% O_2. Experiments were repeated three times independently, with similar results.

(d) The total cell numbers of hPGHESC-1 cells during cell passaging under 5% O_2 and 21% O_2.

(e) The efficiency of deriving mouse haploid ESC lines using human ESC culture medium under 5% O_2 (3/5) or 21% O_2 (2/12) conditions.

(f) One stable hAG-haESC line (termed hAGHESC-1) was generated after two rounds of FACS enrichment of haploid cells.

(g) G-banding analysis of the hAGHESC-2 revealed the normal haploid complement of 23 chromosomes (22+X).

(h) CNV analysis of two hAG-haESC lines (passage 15) showing no major genomic alternations in human haploid cells. Each chromosome is shown in different color.

(i) The total cell numbers of hAGHESC-1 during cell passaging under 5% O_2 or 21% O_2.

(j) The cell cycle analysis of hAGHESC-1 cultured under 5% O_2 or 21% O_2. Cells were stained with Ki67 and Phospho-Histone H3 (Ser10) to distinguish cells in G1/S, G2 or M phase. G1/S-phase cells show two or more separated Ki-67 positive spots; G2-phase cells show one Ki-67 positive foci; and M-phase cells are Ser10 positive.

(k) BrdU pulse-labeling analysis uncovered that fewer cells entered S phase under 5% O_2 compared with 21% O_2. The percentages are BrdU positive and negative ($BrdU^+$ and $BrdU^-$) cells among the Ki-67 positive cells. Two-tailed student's *t* test. ****, $P < 0.0001$.

(l) Summary of hAG-haESC derivation.

Figure S2. Haploidy and Pluripotency of hAG-haESCs.

(a) STR analysis of two hAG-haESC lines and their sperm and oocyte donors.

(b) Expression of human ESC markers in two hAG-haESC lines, including OCT4 (POU5F1), SOX2, SSEA4, TRA-1-60 and TRA-1-81. Scale bars, 50 μm.

(c) Haploidy analysis of two hAG-haESCs (passage 17) through DNA FISH of X chromosome. Magnifications show the haploid nucleus. Green, human chromosome X (ChrX) probe; Blue, nuclear staining with DAPI. Scale bars, 10 μm.

(d) Expression of maker genes of cells from three germ-layers in EBs differentiated from hAG-haESCs, including α-TUJ (ectoderm), α-SMA (mesoderm) and AFP (endoderm). Scale bars, 50 μm.

(e) Histological analysis of teratomas derived from two hAG-haESCs revealed tissues from all three embryonic germ-layers. Scale bars, 50 μm.

(f) FACS analysis of haploid cells in teratomas derived from hAGHESC-1 cells.

(g) DNA FISH of ChrX (green) and immunofluorescence (IF) of AFP (red) in EBs show one X chromosome in differentiated cells. DAPI (blue). Scale bar, 20 μm.

(h) Histological image of teratoma section derived from hAGHESC-2 (left, scale bar, 100 μm). Red box is magnified in Middle showing neuroepithelium (middle, scale bar, 20 μm). Right, a representative DNA FISH image, scale bar, 20 μm. ChrX, green; DAPI, blue.

Figure S3. Imprinted state in human haESCs.

(a) Principal components analysis (PCA) plot showing the gene expression pattern of different samples. Variation values of PC1 and PC2 are 29.27% and 15.08%, respectively. Different samples are shown in different colors, and biology replications are shown in same colors.

(b) Gene expression profiles of human sperm- and oocyte-originated haESCs using RNA-seq analysis. Gene expression profiles were clustered based on known imprinted genes.

(c) PCA plot shows the DNA methylation pattern of different samples. Variation values of PC1 and PC2 are 19.28% and 8.67%, respectively. Different samples are shown in different colors, and biology replications are shown in same colors.

Figure S4. Methylation analysis of typical imprinted genes in hAG-haESCs and their differentiated cells.

(a) DNA methylation state of paternally imprinted regions (*H19* DMR and *IG* DMR) and maternally imprinted region (*SNRPN* DMR) in hAG-haESCs at different passages.

(b) DNA methylation levels at DMRs of paternally and maternally imprinted genes in EBs and teratomas differentiated from hAG-haESCs.

Figure S5. Characterization of reconstructed embryos derived by injection of hAG-haESC into oocytes.

(a-c) *t*-SNE plots showing gene expression patterns of *NANOG* in **(a)**, *GATA4* in **(b)**, and *GATA2* in **(c)** in single cells dissociated from the ICAHCI and ICSI blastocysts according to scRNA-Seq results. Each point represents a single cell sample.

(d-f) *t*-SNE based on all long non-coding RNA (lncRNA) genes showing unbiased clustering results of single cells dissociated from ICAHCI and ICSI blastocysts. Cells are colored based on cell types (**d**), experiment groups (**e**) and embryos (**f**), respectively. ICSI_E8a/b in (**f**) represent two ICSI embryos generated with the oocytes and sperm from the same pair of donors.

(g) Expression of human ESC markers in ICA1 and ICA2 cells, including OCT4 (POU5F1), SOX2, SSEA4, TRA-1-60 and TRA-1-81. Scale bars, 50 μm.

(h) Expression of maker genes of cells from three germ-layers in EBs differentiated from ICA1 and ICA2, including α-TUJ (ectoderm), α-SMA (mesoderm) and AFP (endoderm). Scale bars, 50 μm.

(i) Histological analysis of teratomas derived from ICA1 and ICA2 reveals tissues from all three embryonic germ-layers. Scale bars, 100 μm.

(j) CNV analysis of ICA1 and ICA2 (passage 7) showing no major genomic alternations in human diploid cells derived from ICAHCI blastocysts. Each chromosome is shown in different color.

(k) DNA methylation levels at DMRs of paternally and maternally imprinted genes in teratomas differentiated from ICA1 and ICA2.
