# Supplementary material for: In vitro expansion of human sperm through nuclear transfer
Source: Cell Res. 2019 Dec 18;30(4):356–9. doi: 10.1038/s41422-019-0265-1 (PMC7118075; doi:10.1038/s41422-019-0265-1)
Supplement: Supplementary file 3 — Supplementary information, tables [file 41422_2019_265_MOESM3_ESM.pdf]

Supplementary information.Tables

Table S1. Embryos generated in this study.

Development Stage	Androgenesis	ICSI	ICAHCI
Oocyte (IVM)	256	67	130
ICSI	256	67	-
Enucleation	209	-	-
Pronuclear stage	209*	56#	110#
Two-cell stage	152	38	55
Four-cell stage	98	36	34
Eight-cell stage	95	31	31
Morula	41	16	23
Blastocyst	22	12¶	10¶¶
Outgrowth	13	2	3
ESC line	10	2	2
Haploid ESC line	2	-	-

*: 1PN embryos;

#: 2PN embryos;

¶: 3 out of 12 ICSI blastocysts were used for deriving hESC lines;

¶¶: 4 out of 10 reconstructed blastocysts were used for deriving hESC lines.

Table S2. STR analysis of six ICAHCI embryos used for single-cell RNA sequencing and two diploid ESCs derived from ICAHCI embryos.

STR	hAGHESC-2	ICAHCI-3		ICAHCI-4		ICAHCI-7		ICAHCI-8		ICAHCI-9		ICAHCI-11		ICA1		ICA2	
D3S1358	15	ND	ND	ND	ND	15	14	ND	ND	15	17	ND	ND	15	16	15	15
D6S1043	18	ND	ND	ND	ND	ND	ND	ND	ND	ND	ND	18	17	18	11	18	10
D13S317	12	12	11	12	12	12	10	11	11	13	9	12	10	12	9	12	11
Penta E	11	ND	ND	11	11	11	15	11	20	11	16	11	12	11	17	11	11
D16S539	9	ND	ND	9	10	9	8	ND	ND	9	12	ND	ND	9	9	9	13
D18S51	14	14	16	14	16	14	13	14	22	14	15	14	15	14	18	14	13
D2S1338	23	23	17	23	20	23	23	23	19	23	23	23	24	23	19	23	23
CSF1PO	11	11	12	11	9	11	10	ND	ND	11	12	11	12	11	12	11	13
Penta D	9	9	9	9	13	9	12	9	12	9	9	9	9	9	12	9	9
THO1	9	9	10	9	7	ND	ND	ND	ND	9	8	9	8	9	8	9	9
vWA	18	ND	ND	ND	ND	ND	ND	ND	ND	ND	ND	ND	ND	18	14	18	19
D21S11	29	29	29	29	29	29	31.2	29	29	29	32.2	29	32.2	29	31	29	30
D7S820	11	ND	ND	ND	ND	ND	ND	11	8	ND	ND	11	11	11	8	11	11
D5S818	11	ND	ND	ND	ND	ND	ND	ND	ND	11	10	ND	ND	11	10	11	10
TPOX	11	11	11	11	12	ND	ND	11	11	11	11	ND	ND	11	9	11	11
D8S1179	13	ND	ND	13	13	13	12	13	12	ND	ND	ND	ND	13	13	13	14
D12S391	20	ND	ND	ND	ND	ND	ND	ND	ND	ND	ND	ND	ND	20	20	20	18
D19S433	14.2	ND	ND	14.2	15	14.2	13	ND	ND	14.2	13.2	ND	ND	14.2	13	14.2	12
FGA	20	20	20	20	22	20	20.2	20	22	20	22	20	21	20	23	20	21
	X	X	X	X	X	ND	ND	X	X	ND	ND	ND	ND	X	X	X	X

Table S3. PGS results of the blastocysts used for sc-RNA sequencing

Embryo ID	PGS result
ICAHCI_3	Balanced, Euploid
ICAHCI_4	XO; del(mosaic)(11)(q13.3-q22.1)(32.10Mb)(43%)
ICAHCI_7	Balanced, Euploid
ICAHCI_8	Balanced, Euploid
ICAHCI_9	Balanced, Euploid
ICAHCI_11	Balanced, Euploid
ICSI E3	Balanced, Euploid
ICSI E4	Balanced, Euploid
ICSI E5	Balanced, Euploid
ICSI E6	Balanced, Euploid
ICSI E7	Balanced, Euploid
ICSI E8a	-8
